# Minimal Pruning and Reduced Plant Protection Promote Predatory Mites in Grapevine

**DOI:** 10.3390/insects8030086

**Published:** 2017-08-18

**Authors:** Theresa Pennington, Christian Kraus, Ekatarina Alakina, Martin H. Entling, Christoph Hoffmann

**Affiliations:** 1 Institute for Environmental Sciences, University of Koblenz-Landau, Fortstraße 7, 76829 Landau, Germany; alak1989@uni-landau.de (E.A.); entling@uni-landau.de (M.H.E.); 2 Julius Kühn Institute, Federal Research Institute for Cultivated Plants, Institute for Plant Protection in Viticulture, Geilweilerhof, 76833 Siebeldingen, Germany; christian.kraus@julius-kuehn.de (C.K.); christoph.hoffmann@julius-kuehn.de (C.H.)

**Keywords:** viticulture, beneficial arthropods, *Typhlodromus pyri*, fungicide, fungus-resistant cultivars, sustainable agriculture, ecosystem services, natural pest control

## Abstract

Improving natural pest control by promoting high densities of predatory mites (Acari: Phytoseiidae) is an effective way to prevent damage by pest mites (e.g., Eriophyidae, Tetranychidae) and other arthropod taxa that can cause serious damage to vineyards. Here, we investigate the influence of innovative management on predatory mite densities. We compare (i) full versus reduced fungicide applications and (ii) minimal pruning versus a traditional trellis pruning system in four fungus-resistant grapevine varieties. As predatory mites also feed on fungus mycelium, we assessed fungal infection of grapevine leaves in the experimental vineyard. Predatory mites were significantly more abundant in both minimal pruning and under reduced plant protection. Increases in predatory mites appeared to be independent of fungal infection, suggesting mostly direct effects of reduced fungicides and minimal pruning. In contrast to predatory mites, pest mites did not increase under innovative management. Thus, conditions for natural pest control are improved in fungus-resistant grapevines and under minimal pruning, which adds to other advantages such as environmental safety and reduced production cost.

## 1. Introduction

Growing consumer demand urges agriculture to adopt more sustainable and environmentally friendly practices [1,2]. At the same time, farmers need to secure productivity to satisfy the market. Intensification of farming should take advantage of natural regulation and promote ecosystem services to be sustainable [3]. Viticulture sets a positive example for sustainable farming with the innovation and adaption of cultivars that are resistant to fungal pathogens. They can be a valuable tool to allow for a sustainable intensification, especially when combined with minimal pruning. Planting of these new cultivars can reduce the amounts of sprayed fungicides significantly [4] and minimal pruning increases the yield while still producing high quality grapes [5]. The use of resistant cultivars and minimal pruning may affect phytoseiid mites that play a major role in the biocontrol of pest mites and other arthropods in grape vineyards [6,7,8]. These predatory mites are generalists that can sustain stable populations even when prey numbers are low by surviving and reproducing on alternative food sources such as pollen and fungi [7,9,10,11]. This is an advantage of generalist predators over specialists, whose populations fluctuate depending on prey availability [12]. In addition to food availability, the presence of these beneficial predatory mites in grape vineyards is limited by their susceptibility to pesticides [13,14,15,16]. Once their importance for the agroecosystem was understood, many plant protection chemicals were selected to be less harmful for predatory mites. Despite these measures, some plant protection chemicals are still damaging to mite populations, including the combination of sulfur and copper [17,18] that are both intensively used to control plant pathogenic fungi in organic vineyards. In addition to the plant protection regime, canopy management is another vital part of grapevine production. The pruning system has an important impact on canopy architecture and microclimate [19] and consequently on the arthropod fauna. The traditional vertical shoot positioning (VSP) system and semi-minimal pruned hedge (SMPH) differ in their structural diversity with higher percentage of old wood and more but smaller leaves in SMPH than the VSP grapevines [19]. More structurally complex ecosystems have been shown to enable higher densities of predatory arthropods in both natural and agricultural systems [20] but may impede the host or prey finding success of parasitoids and predators [21,22,23].

We hypothesize that the populations of predatory mites will benefit from a reduced plant protection regime. We expect a higher density of phytoseiids in plots with reduced plant protection compared to those plots that were treated with the fungicide regime typical of fungus susceptible varieties. While pest mites themselves may also benefit from reduced fungicide treatments [17], they should be suppressed by higher densities of phytoseiids. Correspondingly, we expect no benefit or even decreased abundance of pest mites under reduced plant protection. We also hypothesize that the SMPH grapevines offer a more favorable habitat to many arthropods including phytoseiid mites because there is more shelter, better overwintering conditions and a larger, more heterogenous habitat. We investigate these two hypotheses in a controlled field experiment comparing VSP and SMPH pruning regimes under three levels of crop protection intensity.

## 2. Materials and Methods

To analyze the effects of pruning system and plant protection on mite populations in grape vineyards, we used a randomized block design. All data were collected during the two years, 2015 and 2016, in a 15-year-old experimental vineyard of Geilweilerhof located in Siebeldingen, Germany (49°13′13.7″ N, 8°02′43.0″ E). It was planted with four fungus-resistant *Vitis vinifera* cultivars; “Reberger”, “Villaris”, “Felicia” and “Gf 84-58-988”. Interrow distance was 2 m and grapevine spacing was 1 m. We treated the four varieties as our four experimental blocks. Each variety was cultivated in six to ten rows, half of which were VSP and half were SMPH trained. For practical reasons, the pruning treatment was not randomly applied but the two pruning treatments were applied to half of each cultivar block. Each treatment block was again divided into three parts that received one of three randomly assigned organic plant protection regimes. This resulted in six combinations of plant protection intensity and pruning system. Each of those treatment combinations was replicated in the four different varieties, resulting in 24 different treatment plots.

An organic spraying regime consisting of two (“low”), four (“reduced”) or twelve (2016) and thirteen (2015) (“standard”) sprayings of Funguran progress^®^ (Spiess-Urania Chemicals GmbH, Hamburg, Germany) (350 g copper per kg), Wettable Sulfur Stulln (agrostulln GmbH, Stulln, Germany) (796 g sulfur per kg) and VitiSan^®^ (BioFa AG, Münsingen, Germany) (995 g potassium bicarbonate per kg) per season (Table S1). This allowed us to apply standardized amounts of active ingredients in the organic spray treatments. Twelve organic spray treatments equal a frequency of plant protection which is commonly applied to vineyards planted with fungus susceptible grape varieties. Although none of the varieties in our experimental vineyards needed the standard spraying regime, we included the schedule for susceptible cultivars as a reference point to be able to compare the mite populations in susceptible cultivars with a standard spraying regime to the mite populations in fungus-resistant cultivars that need less protection. 

### 2.1. Grapevine Leaf Fauna

To assess the mite fauna on grapevine leaves, we followed the protocol introduced by Hill and Schlamp [24]. Twenty-five mature, randomly selected leaves from vines in each of the 24 treatment plots were collected and washed onto a filter paper, where all mites could be counted and identified using a stereomicroscope (Stemi 2000, Zeiss, Jena, Germany). We counted predatory mites as well as the pest mites *Colomerus vitis* (Pagenstecher) and *Calepitrimerus vitis* (Nalepa) (Acari: Eriophyidae). After the mites were washed off, the leaf area was determined using a leaf area meter (Li-COR, Modell 3100 area meter, Lincoln, NE, USA).

We randomly selected 436 adult predatory mites from the filter paper to determine the species using the preparation method described by Krantz [25] and determined them using a microscope with phase contrast (Leica DM 4000 B, Leica Microsystems, Wetzlar, Germany). We took nine overall samples, two in 2015 (one in July and one in September) and seven in 2016 (one per month in June, July and October and two samples per month in August and September). All samples of one year were combined by averaging mite densities across dates. Data were analysed using R [26]. We fitted a linear mixed model with “grapevine variety” as a random effect and “pruning method” and “plant protection intensity” as fixed effects using the nlme package and the multcomp package for multiple comparisons [27,28].

### 2.2. Fungal Disease

Since the availability of fungal material might be a major factor in regulating phytoseiid populations [7], we monitored the fungus affected leaf area (infection level) as well as the percentage of affected leaves (incidence rate). Monitoring of fungal grapevine diseases was done according to the European and Mediterranean Plant Protection Organization (EPPO) guidelines: *Plasmopara viticola* ([Berk. and M.A. Curtis] Berl. and De Toni, 1888) (PP 1/31(3)), *Erysiphe necator* (Schw.) (PP 1/4(4)), *Botrytis cinerea* (Pers.) (PP 1/17(3)). For each training system and variety, 100 grapevine leaves were screened and rated for disease symptoms of the particular fungal pathogen. The score ranged from 0% (no symptoms) to 100% (symptoms on the whole leaf) with a scaling interval of 10%. Additionally, a scoring of 5% was added to the ranking for the assessment of minimal symptoms. Data were analysed using R [26]. We fitted a linear mixed model with “grapevine variety” as random and “pruning method” and “plant protection intensity” as fixed effects using the nlme package and the multcomp package for multiple comparisons [27,28]. 

## 3. Results

*Typhlodromus pyri* (Scheuten) was by far the most common predatory mite species in our sample with 89%, followed by *Paraseiulus soleiger* (Ribaga) (10%) and *Euseius finlandicus* (Oudemans) (1%).

### 3.1. Effects of Reduced Plant Protection

**Figure 1:**
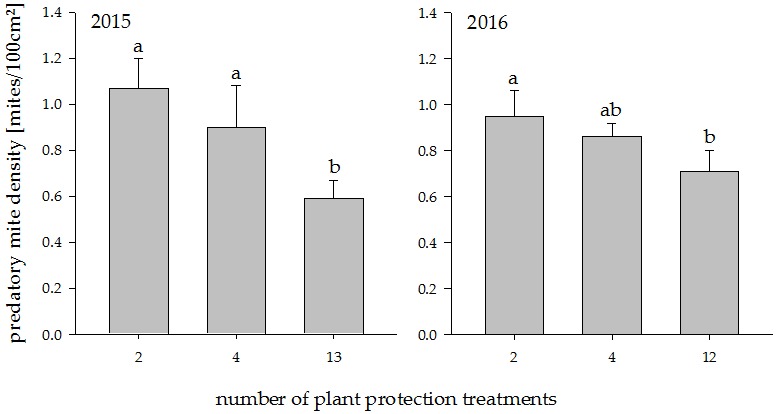
Predatory mite density in plots with increasing plant protection intensities in the years 2015 and 2016. Different letters indicate significant differences between groups (*p* < 0.05). Values represent means + standard error (N = 24).

In both years, reduced plant protection increased the density of predatory mites significantly (Figure 1; 2015: *t*_18_ = −3.80, *p* = 0.0013, 2016: *t*_18_ = −2.39, *p* = 0.028). A post hoc Tukey test showed that in 2015 both lower plant protection treatments differed significantly from the high plant protection treatment at *p* < 0.05. In 2016, the same test revealed significant differences only between 2 and 12 treatments (*p* < 0.05). The difference between the treatments was more pronounced in 2015 than in 2016. In 2015, there were 81% more predatory mites under reduced plant protection than under full plant protection. In 2016, this difference was 34%.

**Figure 2:**
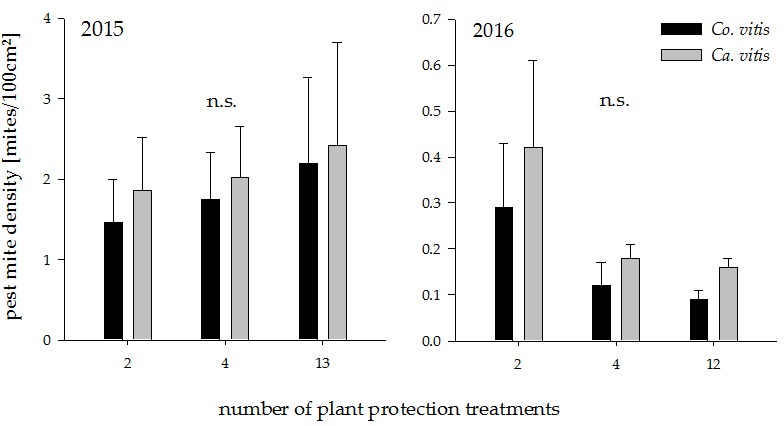
Pest mite density in plots with increasing plant protection intensities in the years 2015 and 2016. n.s. indicates a non-significant difference between groups (*p* > 0.05). Values represent means + standard error (N = 24). Co. = *Colomerus*; Ca. = *Calepitrimerus*.

The densities of the pest mites *Co. vitis* and *Ca. vitis* were low in both years. Plant protection intensity had no significant effect on either pest mite species (Figure 2; 2015: *Co. vitis:* t_18_ = −0.51, *p* = 0.61 *Ca. vitis*: t_18_ = −0.84, *p* = 0.41, 2016: *Co. vitis:* t_18_ = −1.39, *p* = 0.18, *Ca. vitis*: t_18_ = −1.28, *p* = 0.22).

### 3.2. Effects of Minimal Pruning

**Figure 3:**
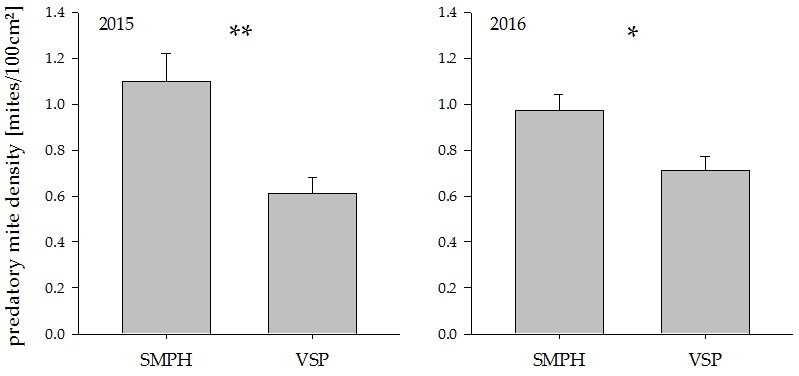
Predatory mite density in minimally pruned versus trellis-trained plots in the years 2015 and 2016. * indicate significant differences between groups (* *p* < 0.05, ** *p* < 0.005). Values represent means + standard errors (N = 24). SMPH: semi-minimal pruned hedge, VSP: vertical shoot positioning.

The predatory mite density was almost two times higher in SMPH compared to VSP grapevines in the year 2015. In the year 2016, the difference was less pronounced but still statistically significant (Figure 3; 2015: *t*_18_ = −4.70, *p* =0.0002, 2016: *t*_18_ = −3.07, *p* = 0.007).

**Figure 4:**
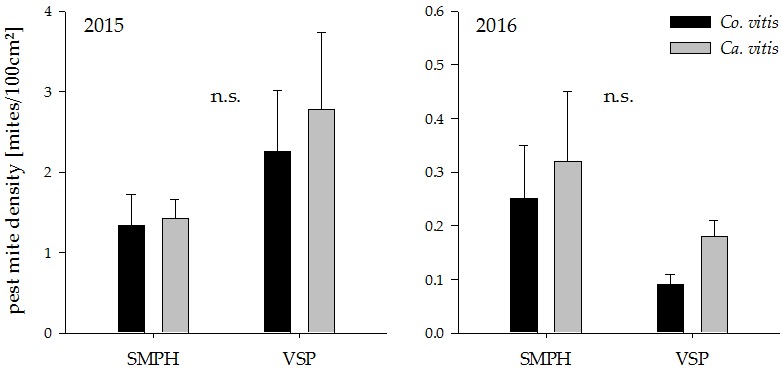
Pest mite density in minimally pruned versus trellis-trained plots in the years 2015 and 2016. n.s. indicates a non-significant difference between groups (*p* > 0.05). Values represent means + standard errors (N = 24). SMPH: semi-minimal pruned hedge, VSP: vertical shoot positioning.

Both pruning systems harbored only low numbers of both observed pest mite species and there was no significant effect of minimal pruning on their density (Figure 4; 2015: *Co. vitis: t*_18_ = 1.28, *p* = 0.21 *Ca. vitis*: *t*_18_ = 1.50, *p* = 0.15, 2016: *Co. vitis: t*_18_ = −1.69, *p* = 0.11, *Ca. vitis*: *t*_18_ = −1.07, *p* = 0.30).

### 3.3. Fungal Disease

We found no infection by *Botrytis cinerea* (botrytis bunch rot) or *Erysiphe necator* (powdery mildew) on grapevine leaves in any of the treatments. There was also no incidence of *Plasmopara viticola* (downy mildew) in 2015 (Table 1). In 2016, we found leaves showing symptoms of *P. viticola* infection all over the vineyard. As expected, higher plant protection intensity had a significant negative effect on *P. viticola* incidence rate and infection levels (incidence rate: *t*_18_ = −5.03, *p* = 0.0001, infection level: *t*_18_ = −3.05, *p* = 0.007). There was also a significantly higher percentage of symptomatic leaves (incidence rate, *t*_18_ = 2.07, *p* = 0.05), although the average affected leaf area (infection level, *t*_18_ = 0.67, *p* = 0.51) was not significantly larger in the SMPH grapevines than in the VSP-trained vines (Table 1).

## 4. Discussion

Minimal pruning and reduced frequency of fungicide sprays enhanced predatory mite abundance in our grape vineyard during both years of the trial. In contrast, densities of pest mites in vineyards under innovative management were generally very low and did not differ significantly between the three fungicide treatment intensities or the two pruning systems. In Europe, both *Ca. vitis* and *Co. vitis* infestations rarely exceed the economic threshold [7]. This is in a large part due to the presence and the active protection of predatory mites such as phytoseiids. Phytoseiid numbers decrease significantly with increasing frequencies and amounts of plant protection applications and there were significantly more mites per leaf area in SMPH-pruned than in VSP-pruned grapevines. The susceptibility of phytoseiid mites to pesticides and fungicides is well described in the literature. For example, Schruft, Wohlfarth and Wegner [15] describe a negative effect on population numbers of *T. pyri* by fungicides containing copper, especially when applied late in the season. They also found a negative influence of sulphur sprayings, but argue that they are not harmful for the population since the effects persisted less than four weeks after the last application. Hanna et al. [29] confirm reduced numbers of *Metaseiulus occidentalis* (Nesbitt) in a vineyard near Madera that was treated with sulfur compared to triadimefon. Hoffmann [17] found that copper and sulfur, in combination, damage up to 80% of the *T. pyri* population when applied frequently in low concentrations as is common practice in organic viticulture.

The negative impact of a combination of sulfur and copper treatments on predatory mite populations in the current study agrees with these earlier studies. Pest mite population numbers were very low in general and there was no significant effect of either of the two management practices. They also benefit from reduced spraying based on the acaricidal properties of sulfur [30,31]. A possible explanation for the lack of a pattern could be that the higher predation pressure from predatory mites, which are more abundant in the reduced fungicide treatments, negates the positive effect of reduced fungicide input on the pest mite population.

Our second hypothesis can be confirmed as well. There were higher densities of Phytoseiids on SMPH-pruned than on VSP-pruned grapevines. One difference between SMPH-pruned and VSP-pruned grapevines are smaller but more abundant leaves in SMPH, resulting in an overall larger leaf surface [19]. This means that there are not only more predatory mites per leaf, but more predatory mites per SMPH vineyard area, increasing the effect even further.

SMPH grapevines probably offer more favorable living conditions for predatory mites. The larger leaf area and the larger amount of wood and especially old wood surface area may offer a higher potential for population growth, better shelter and more oviposition sites. In cracks and crevices of the old wood, mites can find more and better overwintering opportunities, which allow a fast repopulation of the fresh growth in spring and prevents pest mite outbreaks early in the season. Pozzebon et al. [32] describe habitat complexity as a major factor in promoting predatory mite populations.

Although fungal material is an important food source for phytoseiids [7,10], these patterns seem to be independent of the infection of leaves with fungal diseases. Both years showed similar patterns for predatory mite and pest mite densities, even though we detected very different levels of fungal infection between the two years. As expected, in the year 2016, the infection level and the incidence level of *P. viticola* were lower in grapevine that received a more intense plant protection regime. This might amplify the direct negative effect of sulfur and copper on phytoseiids by additionally reducing food availability [18]. The slightly higher infection level with *P. viticola* in the SMPH grapevines can probably be explained by the different microclimate within the canopy. It is more humid and less airy, which promotes fungal growth [19]. Since the population densities of mites were similar in 2015 when there was no incidence of *P. viticola* at all, we assume that this factor is likely contributing to overall predatory mite densities, but is probably overruled by mortality through fungicide application.

It is likely that the more structurally complex SMPH grapevines offer a favorable habitat not only to phytoseiids but also to a number of other species, that are also good alternative food sources for predatory mites in the absence or scarcity of pest mites. It will be interesting to see if the reduced number of sprayings and the minimal pruning method can favor additional beneficials and how the balance of other predator–prey relationships is influenced.

## 5. Conclusions

Reduced fungicide sprayings as well as the more structurally diverse habitat created by minimal pruning both favor beneficial mites, but not pest mites. Thus, minimal pruning of fungus-resistant cultivars improves the conditions for natural pest regulation within the leaf mesofauna of grapevine. Using fungus-resistant cultivars of grapevine and other crops on a larger scale could drastically reduce the frequency of application and thus the impact of plant protection chemicals, not only directly in the crops but also in the surrounding landscape and therefore contribute to the sustainability of agriculture.

## Figures and Tables

**Table 1:** Infection level and incidence rate of *Plasmopara viticola* compared between grapevine leaves under three plant protection intensities and between the pruning methods VSP (vertical shoot positioning) and SMPH (semi-minimal pruned hedge). Significant differences between groups are indicated by different letters.

Year	Measure for Fungal Disease	Number of Plant Protection Treatments			Pruning Method	
		2	4	12/13	VSP	SMPH
2015	infection level ± SE [%]	0	0	0	0	0
	incidence rate ± SE [%]	0	0	0	0	0
2016	infection level ± SE [%]	11.8 ± 2.9 ^a^	5.0 ± 1.2 ^b^	2.5 ± 1.4 ^b^	5.7 ± 1.4	7.1 ± 2.4
	incidence rate ± SE [%]	65.0 ± 7.2 ^a^	49.1 ± 6.6 ^b^	31.8 ± 7.0 ^c^	43.4 ± 8.0 ^a^	53.9 ± 5.1 ^b^

## Supplementary Materials

The following are available online at www.mdpi.com/2075-4450/8/3/86/s1. Table S1: Spraying table.
