# Supplementary material for: Minimal Pruning and Reduced Plant Protection Promote Predatory Mites in Grapevine
Source: Insects. 2017 Aug 18;8(3):86. doi: 10.3390/insects8030086 (PMC5620706; doi:10.3390/insects8030086)
Supplement: Supplementary file 1 [file insects-08-00086-s001.docx]

**Table S1** Spraying table showing the number of treatments as well as the amounts of chemicals used in each treatment for each of the three plant protection intensities (S =‘standard, 12/13 sprayings’, R =‘reduced, 4 sprayings’ and L=‘low, 2 sprayings’) as well as both pruning systems (VSP = vertical shoot positioning, SMPH = semi-minimal pruned hedge) for the years 2015 and 2016.

| 2015 | | | | | | | | | |  |  |  |  |  |  |  |  |  |
| --- | --- | --- | --- | --- | --- | --- | --- | --- | --- | --- | --- | --- | --- | --- | --- | --- | --- | --- |
| VSP | | | | | | | | | SMPH | | | | | | | | | |
| Funguran progress®  pure copper [g/ha] | | | Wettable Stulln  sulfur [kg/ha] | | | VitiSan®  KHCO_3_ [kg/ha] | | | Funguran progress®  pure copper [g/ha] | | | | Wettable Stulln  sulfur [kg/ha] | | | VitiSan®  KHCO_3_ [kg/ha] | | |
| S | R | L | S | R | L | S | R | L | S | | R | L | S | R | L | S | R | L |
| 150 |  |  | 4.5 |  |  |  |  |  | 450 | |  |  | 13.5 |  |  |  |  |  |
| 100 |  |  | 3.6 |  |  |  |  |  | 167 | |  |  | 6.0 |  |  |  |  |  |
| 100 |  |  | 3.6 |  |  |  |  |  | 167 | |  |  | 6.0 |  |  |  |  |  |
| 150 | 150 | 150 | 4.2 | 4.2 | 4.2 |  |  |  | 225 | | 225 | 225 | 6.3 | 6.3 | 6.3 |  |  |  |
| 100 | 100 | 100 | 4.2 | 4.2 | 4.2 |  |  |  | 150 | | 150 | 150 | 6.3 | 6.3 | 6.3 |  |  |  |
| 100 | 100 |  | 4.8 | 4.8 |  |  |  |  | 140 | | 140 |  | 6.7 | 6.7 |  |  |  |  |
| 250 | 250 |  | 3.2 | 3.2 |  |  |  |  | 350 | | 350 |  | 4.5 | 4.6 |  |  |  |  |
| 200 |  |  | 3.2 |  |  |  |  |  | 280 | |  |  | 4.6 |  |  |  |  |  |
| 200 |  |  | 3.2 |  |  |  |  |  | 280 | |  |  | 4.6 |  |  |  |  |  |
| 200 |  |  | 3.2 |  |  |  |  |  | 233 | |  |  | 3.4 |  |  |  |  |  |
| 200 |  |  |  |  |  | 4 |  |  | 233 | |  |  |  |  |  | 4.7 |  |  |
| 200 |  |  |  |  |  | 6 |  |  | 233 | |  |  |  |  |  | 7 |  |  |
| 200 |  |  |  |  |  | 6 |  |  |  | |  |  |  |  |  | 7 |  |  |
| 2016 | | | | | | | | | |  |  |  |  |  |  |  |  |  |
| VSP | | | | | | | | | SMPH | | | | | | | | | |
| Funguran progress®  pure copper [g/ha] | | | Wettable Stulln  sulfur [kg/ha] | | | VitiSan®  KHCO_3_ [kg/ha] | | | Funguran progress®  pure copper [g/ha] | | | | Netzschwefel Stulln  sulfur [kg/ha] | | | VitiSan®  KHCO_3_ [kg/ha] | | |
| S | R | L | S | R | L | S | R | L | S | | R | L | S | R | L | S | R | L |
| 100 |  |  | 3.6 |  |  |  |  |  | 167 | |  |  | 6.0 |  |  |  |  |  |
| 200 |  |  | 3.6 |  |  |  |  |  | 333 | |  |  | 6.0 |  |  |  |  |  |
| 600 | 600 |  | 3.6 | 3.6 |  |  |  |  | 800 | | 800 |  | 5.4 | 5.4 |  |  |  |  |
| 300 | 300 | 300 | 3.6 | 3.6 | 3.6 |  |  |  | 420 | | 420 | 420 | 5.0 | 5.0 | 5.0 |  |  |  |
| 300 | 300 | 300 | 5.0 | 5.0 | 5.0 |  |  |  | 420 | | 420 | 420 | 7.0 | 7.0 | 7.0 |  |  |  |
| 300 | 300 |  | 4.0 | 4.0 |  |  |  |  | 400 | | 400 |  | 5.3 | 5.3 |  |  |  |  |
| 300 |  |  | 4.0 |  |  |  |  |  | 400 | |  |  | 5.3 |  |  |  |  |  |
| 200 |  |  | 4.0 |  |  |  |  |  | 267 | |  |  | 5.3 |  |  |  |  |  |
| 200 |  |  | 4.0 |  |  |  |  |  | 267 | |  |  | 5.3 |  |  |  |  |  |
| 200 |  |  | 4.0 |  |  |  |  |  | 267 | |  |  | 5.3 |  |  |  |  |  |
| 200 |  |  |  |  |  | 5 |  |  | 267 | |  |  |  |  |  | 6.7 |  |  |
| 200 |  |  |  |  |  | 6 |  |  | 267 | |  |  |  |  |  | 8 |  |  |
